# Enhancing Photoluminescence of CsPb(Cl_x_Br_1−x_)_3_ Perovskite Nanocrystals by Fe^2+^ Doping

**DOI:** 10.3390/nano13030533

**Published:** 2023-01-28

**Authors:** Chang Wu, Yan Li, Zhengyao Xia, Cheng Ji, Yuqian Tang, Jinlei Zhang, Chunlan Ma, Ju Gao

**Affiliations:** 1 Jiangsu Key Laboratory of Micro and Nano Heat Fluid Flow Technology and Energy Application, School of Physical Science and Technology, Suzhou University of Science and Technology, Suzhou 215009, China; 2 School of Environmental Science and Engineering, Suzhou University of Science and Technology, Suzhou 215009, China; 3 School Optoelect Engn, Zaozhuang University, Zaozhuang 277160, China

**Keywords:** photoluminescence, perovskite nanocrystals, Fe^2+^ doping

## Abstract

The doping of impurity ions into perovskite lattices has been scrupulously developed as a promising method to stabilize the crystallographic structure and modulate the optoelectronic properties. However, the photoluminescence (PL) of Fe^2+^-doped mixed halide perovskite NCs is still relatively unexplored. In this work, the Fe^2+^-doped CsPb(Cl_x_Br_1−x_)_3_ nanocrystals (NCs) are prepared by a hot injection method. In addition, their optical absorption, photoluminescence (PL), PL lifetimes, and photostabilities are compared with those of undoped CsPb(Br_1−x_Cl_x_)_3_ NCs. We find the Fe^2+^ doping results in the redshift of the absorption edge and PL. Moreover, the full width at half maximums (FWHMs) are decreased, PL quantum yields (QYs) are improved, and PL lifetimes are extended, suggesting the defect density is reduced by the Fe^2+^ doping. Moreover, the photostability is significantly improved after the Fe^2+^ doping. Therefore, this work reveals that Fe^2+^ doping is a very promising approach to modulate the optical properties of mixed halide perovskite NCs.

## 1. Introduction

Lead halide ABX_3_ (X = Cl, Br, I) perovskites are very competitive semiconductors for solar cells, lighting, and photodetectors owing to their tremendous optical properties, such as a tunable fluorescent color across the whole visible range, strong absorption, high photoluminescence (PL) quantum yields (QYs), narrow PL bandwidths, and suppressed PL blinking [1,2,3,4,5,6,7,8]. Although the perovskites exhibit extraordinary potential and there are already many exciting progresses, the further commercialization of these promising semiconductors still confronts severe challenges. One is the inclusion of toxic Pb and another one is the instability against moisture, oxygen, heat, and electric current/irradiation [9,10,11,12,13,14]. In particular, for the mixed halide perovskites, the photo- or electric-induced ion migration is ineluctable, resulting in phase segregation [15,16,17]. Therefore, during the past years, many research works have been conducted to decrease the use of toxic Pb and improve the stability of perovskites [18,19,20]. To avoid the utilization of toxic lead, various lead-free perovskites, such as Sn^2+^, Sn^4+^, Mn^2+^, and Cu^2+^-based perovskites have been exploited [18,19,20]. Among them, tin-based perovskites have been the most explored [19]. However, the Sn^2+^ in perovskite is easily oxidized to Sn^4+^, causing poor stability [21]. The PL QYs of tin-based perovskites and power conversion efficiency (PCE) of solar cells based on tin-based perovskites are very low [11].

Doping of other metal ions to partially replace Pb is another effective way to reduce the utilization of toxic lead, while maintaining or even enhancing the excellent optical and photoelectrical properties. The incorporation of appropriate impurity ions into host lattices has been exploited as a promising method to stabilize the crystallographic phases while modulating the optical, electronic, and magnetic properties of diverse semiconductors [22,23,24,25,26,27,28]. Regarding the halide perovskites, the partial substitution of Pb^2+^ by divalent metal ions, such as Cu^2+^, Mg^2+^, Fe^2+^, Co^2+^, Ni^2+^, and Mn^2+^ at the B sites of the perovskite lattice have been demonstrated [29,30,31,32,33,34,35,36]. Klug et al revealed that the perovskite films retain an excellent photovoltaic performance if less than 3% Pb^2+^ ions are substituted by homovalent metal species due to the high tolerance of the perovskite lattices [31]. To date, many different research groups have recently demonstrated that Mn^2+^ ions can be doped into the B sites of perovskite lattices by using a facile approach [32,33,34,35]. Moreover, a broad PL peak at about 600 nm is induced by the ^4^T_1_ to ^6^A_1_ transition of Mn^2+^, which can be applied for multicolor luminescence [35]. After anion exchange reactions between Mn-doped CsPbCl_3_ and CsPbBr_3_, fluorescence color gamut almost covering the entire visible spectrum are obtained [35]. Thanks to the high stability and wide color gamut, color converters for light-emitting diodes (LEDs) were constructed. Similarly, the doping of Ni^2+^ ions into all inorganic perovskite nanocrystals (NCs) can also modulate the PL. Sun et al have a general strategy for the synthesis of Ni-doped CsPbCl_3_ NCs, which shows a strong single-color violet emission with a maximum PL QY of 96.5% [36].

Fe ions, as the earth-abundant elements, are eco-friendly and low-cost. The doping of Fe ions in perovskites has also attracted research interests. CH_3_NH_3_PbCl_3_ single crystals with different concentrations of Fe^2+^/Fe^3+^ doping were synthesized by Cheng et al [37]. In addition, the crystal structure, optical, and optoelectronic properties were investigated. They revealed that Fe^2+^ is prone to replacing Pb^2+^ and the optoelectronic properties are seriously deteriorated. On the contrary, Hu et al. reported that an appropriate amount of Fe^2+^ doping into the lattice of CsPbCl_3_ NCs not only improved the homogeneity of the size of the NCs, but also enhanced the PL QY and average PL lifetimes [38]. Therefore, the impact of Fe^2+^ doping on the optoelectronic properties of perovskite NCs is still unclear.

In this work, the Fe^2+^-doped CsPb_1−x_Fe_x_(Br_1−x_Cl_x_)_3_ NCs are prepared by mixing FeCl_2_ (x mmol) and PbBr_2_ (1−x mmol) during the hot injection process. When x is not zero, Pb^2+^ at the B sites are partially replaced by Fe^2+^; meanwhile, the X sites are also partially occupied by Cl-. Therefore, the PL properties of Fe^2+^-doped mixed halide perovskite NCs are investigated. To our knowledge, the PL of Fe^2+-^doped mixed halide perovskite NCs is studied for the first time. The morphology and size distribution of the Fe^2+-^doped perovskite NCs are investigated by a transmission electron microscope (TEM). The optical absorption, PL, PL lifetimes, and photostabilities of the Fe^2+^-doped perovskite NCs are measured, which are compared with those of the undoped CsPb(Br_1−x_Clx)_3_ NCs.

## 2. Materials and Methods

### 2.1. Synthesis of CsPb_1−x_Fe_x_(Br_1−x_Cl_x_)_3_ (x = 0, 0.1, 0.2, and 0.3) NCs

Cs-oleate was prepared by dispersing Cs_2_CO_3_ powders (0.407 g, 1.25 mmol) into a mixture of 18 mL octadecene (ODE, Aladdin, 90%) and 1.74 mL oleic acid (OA, Aldrich, 90%), which was then heated to 150 °C under N_2_ atmosphere until all Cs_2_CO_3_ powders were reacted. Then, FeCl_2_ (x mmol, x = 0, 0.1, 0.2, and 0.3) and PbBr_2_ (1−x mmol) were mixed with OLA (1.5 mL), oleylamine (OLA, 70%, 1.5 mL), trioctylphosphine (TOP, 90%, 1 mL), and ODE (10 mL) in a 100 mL 3-neck flask. The mixture was then degassed at 110 °C for 40 min. After that, the mixture was heated to 170 °C under N_2_ atmosphere. After reaction for 15 s, the hot Cs-oleate precursor (1 mL) was quickly injected. Subsequently, an ice-water bath was used to cool down the reaction. The resulting solution was centrifuged at 5000 r/m for 5 min, the supernatant was discarded. To remove the residual reactants, the solids were redispersed in hexane and centrifuged again for 5 min. When x = 0.4, well-shaped perovskite nanocrystals cannot be obtained. The reaction was conducted under protection of N_2_ and no other oxidant was added. Thus, the oxidation of feeding Fe^2+^ to Fe^3+^ is negligible. Moreover, Fe^2+^ is expected to replace Pb^2+^ as confirmed previously [37,38]. It is reasonable that the main valence of doping iron is bivalent.

### 2.2. Characterizations

Transmission electron microscope (TEM) images were captured on the JEM-2100F electron microscope (JOEL, Japan Electronics Co., Ltd, Tokyo, Japan). Elemental analysis was conducted using energy-dispersive X-ray spectroscopy (EDS) coupled on the TEM. The absorption spectra were measured by using a Shimazu UV2600 UV-Vis spectrophotometer, Shimazu Co., Ltd, Tokyo, Japan). The PL spectra were measured by a Maya 2000 Pro high sensitivity spectrometer (Ocean Optics Co., Ltd, Orlando, USA). The time-resolved PL decay curves were measured by a PicoHarp 300 time-correlated single photon counting (TCSPC) system (Pico Quant Co., Ltd, Berlin, Germany). 

## 3. Results and Discussion

### 3.1. Structure

**Figure 1:**
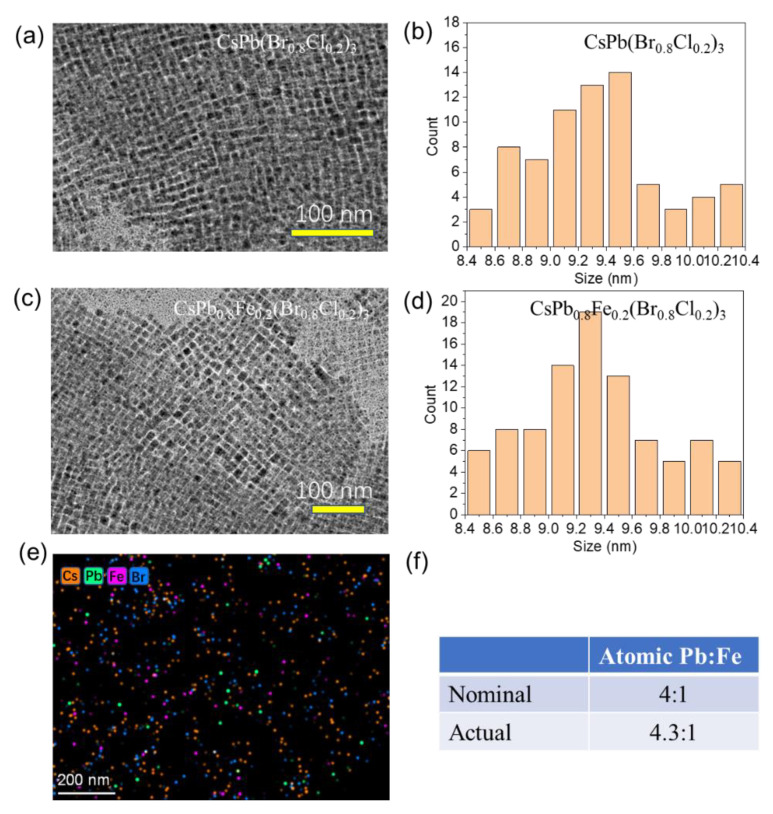
(**a**,**b**) TEM image (**a**) and size distribution (**b**) of the CsPb(Br_0.8_Cl_0.2_)_3_ NCs. (**c**,**d**) TEM image and size distribution of the CsPb_0.8_Fe_0.2_(Br_0.8_Cl_0.2_)_3_ NCs. (**e**) Elemental mappings of the CsPb_0.8_Fe_0.2_(Br_0.8_Cl_0.2_)_3_ NCs. (**f**) Atomic ratio between Pb and Fe of the CsPb_0.8_Fe_0.2_(Br_0.8_Cl_0.2_)_3_ NCs.

Figure 1a,b show the TEM and size distribution of the CsPb(Br_0.8_Cl_0.2_)_3_ NCs without the Fe^2+^ doping. The Fe^2+^-doped perovskite NCs were prepared by a hot injection method. The feeding ratio between the FeCl_2_ (x mmol) and PbBr_2_ (1−x mmol) was adjusted to simultaneously control the doping concentration and halide composition. Figure 1c shows the TEM image of the CsPb_1−x_Fe_x_(Br_1−x_Cl_x_)_3_ NCs when x = 0.2. It shows that the NCs are monodispersed with regular cubic shapes. In addition, a size distribution is obtained based on the TEM image. As shown in Figure 1d, the sizes of the CsPb_0.8_Fe_0.2_(Br_0.8_Cl_0.2_)_3_ NCs are about 9.3 nm with a relatively uniform distribution. The elemental distributions are measured by energy-dispersive X-ray spectroscopy (EDS) equipped on the TEM. The result shown in Figure 1e suggests the homogeneous presence of Fe, indicating the Fe^2+^ ions are successfully doped into NCs. The actual atomic ratio between Pb and Fe determined by the whole EDS mapping of Figure 1e is about 4.3:1 in Figure 1f. Both the shape and size distribution of the CsPb(Br_1−x_Cl_x_)_3_ NCs are very similar to those of the CsPb_1−x_Fe_x_(Br_1−x_Cl_x_)_3_ NCs. Therefore, we found the doping of the Fe^2+^ ion at a low concentration has a negligible influence on the growth of perovskite NCs.

**Figure 2:**
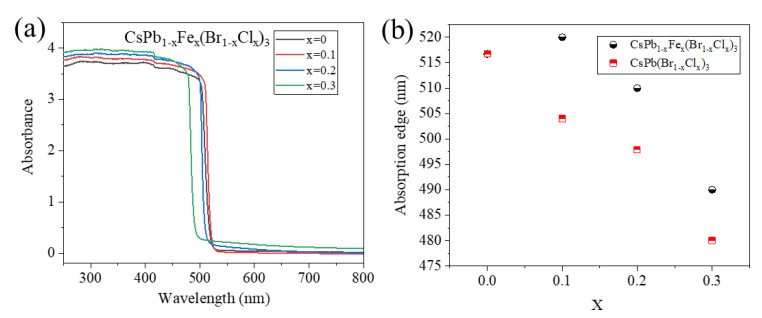
(**a**) Absorption spectra of the CsPb_1*−*x_Fe_x_(Br_1*−*x_Cl_x_)_3_ NCs. (**b**) Absorption band edge positions of the CsPb_1*−*x_Fe_x_(Br_1*−*x_Cl_x_)_3_ NCs at different x. Absorption band edge positions of the CsPb_1*−*x_Fe_x_(Br_1*−*x_Cl_x_)_3_ are also plotted for comparison.

The absorption spectra of the CsPb_1−x_Fe_x_(Br_1−x_Cl_x_)_3_ NCs are shown in Figure 2a. All the absorption spectra show a sharp edge with a negligible Urbach tail, indicating the low density of defect trapping. When x is not zero, Pb^2+^ at the B sites are partially replaced by Fe^2+^; meanwhile, the X sites are also partially occupied by Cl^−^. Typically, as the fraction of Cl^−^ increases, the bandgap of the perovskite increases and the PL peak position blueshifts [39]. The relationship between the absorption edge position and x value is presented in Figure 2b. Surprisingly, the absorption edge of the CsPb_0.9_Fe_0.1_(Br_0.9_C_l0.1_)_3_ NCs redshift slightly compared to that of the CsPbBr_3_. As x increases from 0 to 0.3, the absorption edge slightly redshifts first and then blueshifts. Mixed halide CsPb(Br_1−x_Cl_x_)_3_ perovskite NCs without the Fe doping are also synthesized by mixing PbCl_2_ (x mmol) and PbBr_2_ (1−x mmol) during the preparation. The absorption edge positions of the CsPb(Br_1−x_Cl_x_)_3_ NCs without the Fe^2+^ doping are added for comparison. The absorption edge position gradually blueshifts as x increases. All the absorption edges of the Fe^2+^-doped NCs redshift compared to those of the corresponding perovskite NCs without the Fe^2+^ doping.

### 3.2. Photoluminescence Properties

**Figure 3:**
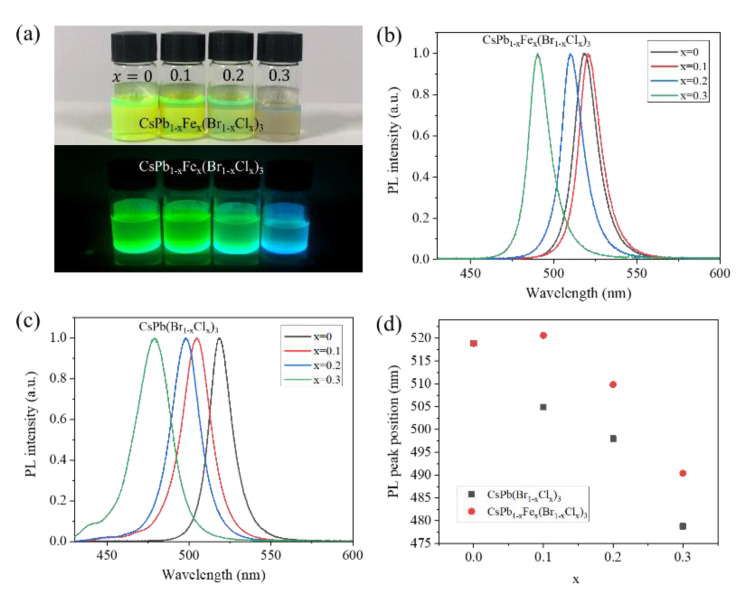
(**a**) Photographs of CsPb_1−x_Fe_x_(Br_1−x_Clx)_3_ NC solutions under natural light (top) and UV light (bottom). (**b**) Normalized PL spectra of CsPb_1−x_Fe_x_(Br_1−x_Cl_x_)_3_ NC solutions. (**c**) Normalized PL spectra of CsPb(Br_1−x_Cl_x_)_3_ NC solutions. (**d**) Comparison of PL peak positions of CsPb_1−x_Fe_x_(Br_1−x_Cl_x_)_3_ and CsPb(Br_1−x_Cl_x_)_3_ NC solutions at different x (x = 0, 0.1, 0.2, 0.3). The excitation wavelength kept as 405 nm.

Photos of the CsPb_1−x_Fe_x_(Br_1−x_Cl_x_)_3_ NC solutions under natural daylight and UV light are shown in Figure 3a. As x changes from 0 to 0.3, the fluorescent color turns from green to cyan. The corresponding PL spectra of the CsPb_1−x_Fe_x_(Br_1−x_Cl_x_)_3_ NCs with x = 0 to 0.3 are displayed in Figure 3b. For x = 0, the PL peak position of the CsPbBr_3_ NCs appears at about 519 nm, which is in good agreement with previous work. Similar to the shift of the absorption edge, when x = 0.1, the PL peak position of the CsPb_1−x_Fe_x_(Br_1−x_Cl_x_)_3_ NCs redshifts to 521 nm. As x increases to 0.2 and 0.3, the PL peak positions apparently blueshift. When x = 0.3, the peak position appears at 490 nm, explaining the cyan fluorescent color. The PL spectra of the CsPb(Br_1−x_Cl_x_)_3_ NCs without the Fe^2+^ doping are shown in Figure 3c. The PL peak position monotonously blueshifts as the x value increases without any exception. The dependencies of the PL peak position on the x value for these two groups of perovskite NCs are plotted in Figure 3d. For the same x value, the PL peak position of the CsPb_1−x_Fe_x_(Br_1−x_Cl_x_)_3_ NCs always redshifts compared to that of the CsPb(Br_1−x_Cl_x_)_3_ NCs, which is again in good agreement with the shift of the absorption edge. Therefore, we can conclude that the Fe^2+^ doping results in the redshift of PL. 

Many different effects can be responsible for the PL redshift. For example, the large size means a narrowed bandgap due to the size effect, which can explain the PL redshift [39]. Moreover, photon reabsorption is another possible reason for the PL redshift [40,41]. As revealed previously [41], evident photon reabsorption prevailingly exists in various halide perovskite materials due to their high absorption coefficient and small Stokes shift. The Fe^2+^ doping may increase the aggregation of NCs, leading to the PL redshift. However, the TEM images do not show either an increased size or increased aggregation, excluding these factors. In addition, the element doping may expand the lattice spacings, causing the shrinkage of the bandgap. However, here, the doping element is Fe^2+^ with a radius of ~0.76 Å, which is smaller than the Pb^2+^ with a radius of ∼1.33 Å. In fact, previous work has reported that the doping of Fe^2+^ in CsPbCl_3_ NCs results in a slight blueshift of the PL peak position [38]. Furthermore, the surface defects generally induce shallow traps, leading to a narrowing of the bandgap. The Fe^2+^ doping may induce shallow traps in the perovskite lattices, which leads to the PL redshift. This kind of possibility is evaluated by our further investigation.

**Figure 4:**
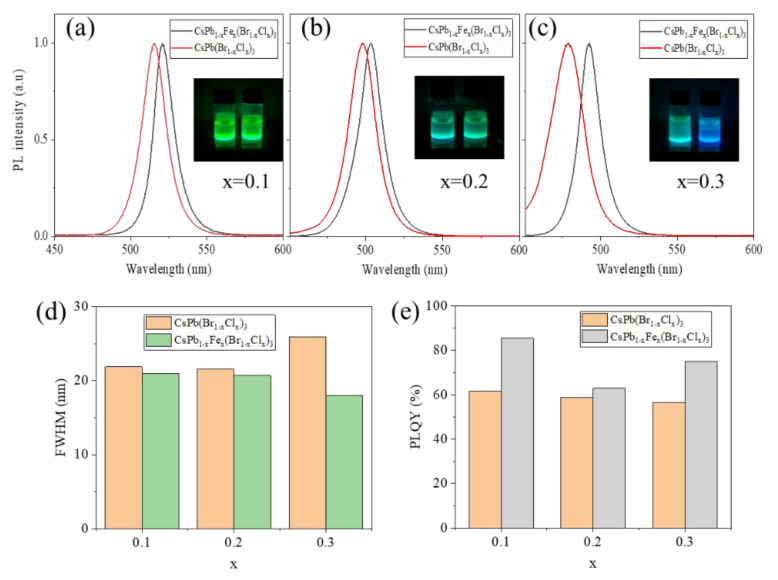
(**a**–**c**) Comparison of PL spectra of the CsPb_1−x_Fe_x_(Br_1−x_Cl_x_)_3_ and CsPb(Br_1−x_Cl_x_)_3_ NC solutions with x = 0.1 (**a**), 0.2 (**b**), and 0.3 (**c**). (**d**) FWHM of the two kinds of NCs with different x values. (**e**) PL QY of the two kinds of NCs with different x values.

The PL spectra of the CsPb_1**−**x_Fe_x_(Br_1**−**x_Cl_x_)_3_ NCs and CsPb(Br_1**−**x_Cl_x_)_3_ NCs are separately compared in Figure 4a–c for x = 0.1 to 0.3. It can be clearly seen that, not only the PL peak position redshifts but also the bandwidth decreases in the Fe^2+^-doped samples. The full widths at half maximums (FWHMs) are compared in Figure 4d. Especially when x = 0.3, the FWHM of the PL of the CsPb(Br_1**−**x_Cl_x_)_3_ NCs is about 26 nm, which decreases to about 18 nm after the Fe^2+^ doping. The PL bandwidth depends on both intrinsic effects, such as electron–phonon interactions, shallow defect states, and extrinsic factors, such as size polydispersity. Herein, the size distribution is little changed by the Fe^2+^ doping. Therefore, the reduced FWHM is mainly attributed to the intrinsic effects. It is well supposed that both the doping of Fe^2+^ improves the crystallinity and diminishes the defect states. To verify this point, the PL QYs of the perovskite NCs before and after the Fe^2+^ doping were measured and compared. As shown in Figure 4e, the PL QYs are improved after different concentrations of the Fe^2+^ doping. Therefore, both the FWHMs and PL QYs suggest the density of defects is reduced by the appropriate Fe^2+^ doping.

**Figure 5:**
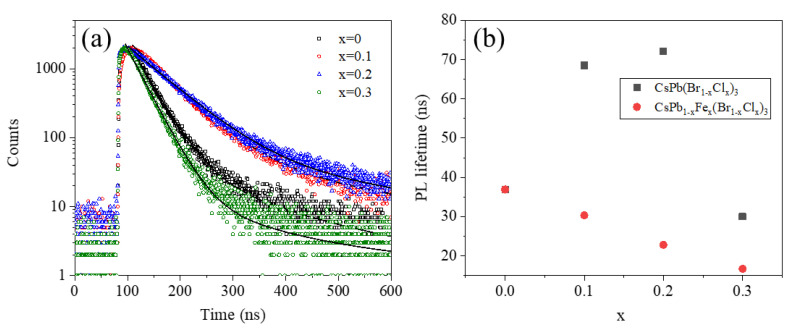
(**a**) Time-resolved PL spectra of the CsPb_1−x_Fe_x_(Br_1−x_Cl_x_)_3_; (**b**) PL lifetime parameters of the CsPb_1−x_Fe_x_(Br_1−x_Cl_x_)_3_ obtained from numerical fitting on (**a**). PL lifetimes of the CsPb(Br_1−x_Cl_x_)_3_ are also plotted for comparison.

To further understand the PL of the CsPb_1-x_Fe_x_(Br_1**−**x_Cl_x_)_3_ NCs, the time-resolved PL decay spectra were measured. As shown in Figure 5a, all the time-resolved PL decays are fitted by an exponential function. The PL lifetimes are shown in Figure 5b. The PL lifetimes of the undoped CsPb(Br_1–x_Cl_x_)_3_ NCs are also added for comparison. The PL lifetimes of the undoped CsPb(Br_1−x_Cl_x_)_3_ monotonously decrease as x increases, which has been widely reported previously [39]. However, for the CsPb_1−x_Fe_x_(Br_1−x_Cl_x_)_3_ NCs, the PL lifetimes increase to about 70 ns when x = 0.1 and 0.2, which are significantly longer than those of the undoped CsPb(Br_1−x_Cl_x_)_3_. The time-resolved PL decay spectra indicate the PL lifetimes of the mixed halide perovskite NCs are increased by the Fe doping. The defect trapping usually leads to a short PL lifetime [42,43]. Thus, a longer PL lifetime implies a lower defect density [42,43]. Therefore, the PL lifetime further suggests the defect density is decreased after the Fe^2+^ doping, which is in good agreement with the decreased FWHMs and improved PL QYs. Back to the PL peak positions shown in Figure 3, the redshift of the emission cannot be attributed to the shallow traps caused by the Fe^2+^ doping.

**Figure 6:**
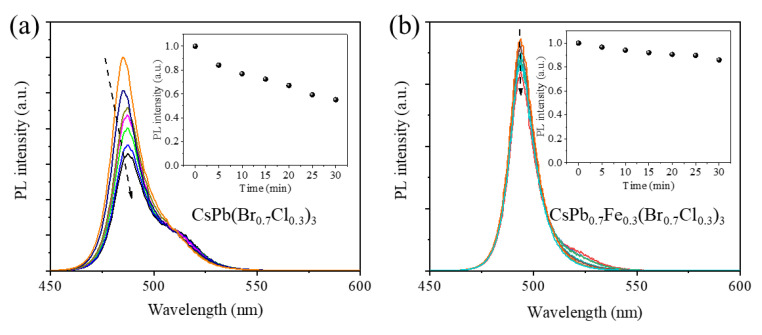
(**a**) PL spectra of CsPb(Br_1−x_Cl_x_)_3_ NCs under continuous UV irradiation. (**b**) PL spectra of CsPb_1−x_Fe_x_(Br_1−x_Cl_x_)_3_ NCs under continuous UV irradiation. Insets: PL intensity versus irradiation time.

As is well known, the photostability of the CsPb(Br_1−x_Cl_x_)_3_ NCs is generally worse than that of the CsPbBrCl_3_ NCs due to the phase segregation [15,16,17]. After continuous irradiation, the mixed CsPb(Br_1−x_Cl_x_)_3_ NCs transform to a separated Br rich phase and Cl rich phase, and the PL shows typical redshifts and quenches largely. As shown in Figure 6a, the PL intensity of the CsPb(Br_1−x_Cl_x_)_3_ NCs quenches to about half of the original intensity after 1 W UV irradiation for 30 minutes. After the Fe^2+^ doping, we find the photostability is significantly improved. The PL intensity only decreases by ca.15% after UV irradiation for 30 minutes. The doping of the appropriate concentration of Fe^2+^ (∼0.76 Å) with an ionic radius smaller than Pb^2+^ (∼1.33 Å) can enhance the formation energies of perovskite lattices and, thus, essentially improve the structural stability [44,45]. Moreover, the stability usually depends on the crystal quality. The improved photostability is consistent with the decreased defect density.

## 4. Conclusions

In summary, the Fe^2+^-doped perovskite NCs are prepared by a hot injection method. In addition, their optical properties, including absorption, PL, and PL lifetimes are compared with those of the undoped CsPb(Br_1−x_Cl_x_)_3_ NCs. We find that Fe^2+^ doping results in the redshift of the absorption edge and PL. Moreover, the FWHMs are decreased and PL QYs are improved by the Fe^2+^ doping, suggesting the density of defects is reduced. The extended PL lifetimes further verify the defect density is decreased after the Fe^2+^ doping. Moreover, the photostability is significantly improved after the Fe doping. Therefore, this work reveals that Fe^2+^ doping is a very promising approach to modulate the optical properties of mixed halide perovskite NCs. 

## Data Availability

All the experimental/calculation date that support the findings of this study are available from the corresponding authors upon reasonable request.
